# Epidemiological and Clinical Aspects and Therapy of Chronic Otitis Media in the “ENT” and Cervicofacial Surgery Ward in the University Hospital of Ouagadougou

**DOI:** 10.1155/2013/698382

**Published:** 2013-08-26

**Authors:** Y. M. C. Gyebre, R. W.-L. Ouedraogo, A. Elola, B. P. Ouedraogo, M. Sereme, M. Ouattara, K. Ouoba

**Affiliations:** ^1^Department of Otolaryngology (ENT) and Cervico-Facial Surgery (CFS), “Yalgado Ouedraogo” University Hospital (CHU-YO), 03 BP 7022, Ouagadougou 03, Burkina Faso; ^2^Faculty of Health Sciences (FHS), 03 BP 7021, Ouagadougou 03, Burkina Faso; ^3^Department of ENT and CFS, “Souro Sanou ” Hospital (CHU-SS), Bobo-Dioulasso, Burkina Faso

## Abstract

*Objectives*. The aim of this study was to analyze the epidemiological and clinical aspects of chronic otitis media and its therapeutic processes in our context. *Patients and Methods*. In a prospective study over a period of 1 year (March 2009–February 2010), 79 patients with chronic otitis media have been cared for in the otolaryngology ward of the University Hospital of Ouagadougou. *Results*. Chronic otitis media (COM) commonly occurs in the age group from 0 to 15 years (40.50%). Otorrhea was the main reason for consultation in 53 cases (67.10%); the most frequently encountered clinicopathological forms were simple COM (71%) followed by otitis media with effusion (24.30%). Intra-auricular instillations of traditional products (46.09%) were the dominant favoring factor. Treatment was essentially through medication in 59 cases with a stabilization of lesions. Endotemporal complications were noticed in 6 cases. *Conclusion*. The fight against chronic otitis media is carried out through preventive measures of education the of people.

## 1. Introduction

Chronic otitis media (COM) remains a frequent pathology. Its evolution can run through serious complications and irreversible damage [1, 2], mainly in our areas which are characterized by low medication and delays in referral specialist consultations. The issue of the COM in developing countries still lies not only in its diagnostic approaches, but also in its treatment modalities.

The purpose of this study was to analyze the epidemiological, clinical, and therapeutic aspects of this disease at the University Hospital of Ouagadougou.

## 2. Patients and Methods

 This prospective descriptive study focused on 79 patients with COM, aged from 6 months to 75 years. These cases have been collected from March 1 2009, to February 29 2010, in the ENT ward of the University Hospital (CHU-YO) of Ouagadougou.

Eight hundred and fifty ears were examined during the study. It included all patients with COM who came in consultation during the said period in the service and had given their informed consent. The diagnosis of COM was based upon the following arguments: chronic inflammation of the middle ear lasting for more than 3 months with otorrhea or not, and changes in the eardrum, eardrum perforation or not, pure tone threshold audiometry showing a hearing loss. Patients had a follow-up control check on the 15th, 30th, 60th, and 90th days after treatment. Controls evaluated local changes (ending of otorrhea, aspect of the eardrum, and the bottom case, with research of complications).

We developed a data collection sheet taking into account age, gender, history, datasheet of the clinical examination, further investigations, treatment, and evolution.

## 3. Results

### 3.1. Epidemiological Data

**Figure 1:**
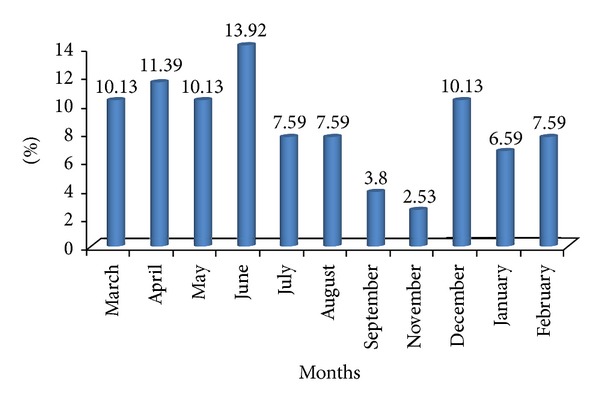
Distribution of patients per month of consultation (*n* = 79).

Seventy-nine patients (0.96%) were recruited during the study period among a group of 8–200 patients who had consulted. The seasonal incidence of COM has been shown in Figure 1.

There were 43 men (54.3%) and 36 women (45.70%) with a sex ratio of 1.19. The average age was 21 years with extremes 6 months and 75 years.

The most common age was that from 0 to 15 years with a frequency of 32 cases or 40.50% of cases.

### 3.2. Clinical Data

The average consultation time was 6 months. Twenty-one patients or 25% had had their first special consultation after more than 10 months of disease development. The main reasons for consultation were otorrhea, 53 cases (67.10%), followed by earache, 13 cases (16.50%), and hearing loss, 8 cases (10.13%).

ENT histories found in our patients were listed in Table 1.

Contributing factors found in our patients were shown in Table 2.

**Figure 2:**
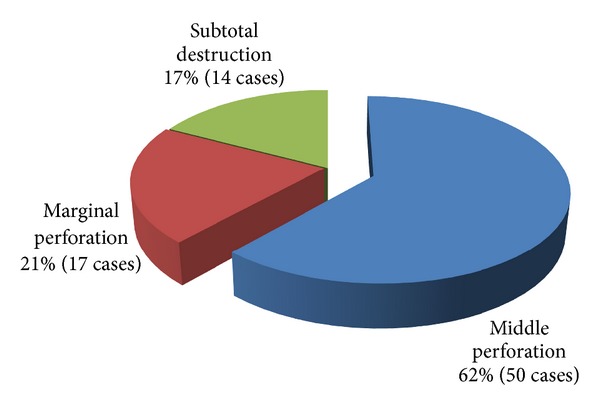
Distribution of cases of tympanic perforation by site (*n* = 81).

Bilateral COM (35.40%) was relatively more common than unilateral shapes taken as isolated cases: right (30.40%) and left (34.20%). Among the 158 ears examined, 107 showed COM. The distribution of tympanic perforations according to location has been represented in Figure 2.

**Figure 3:**
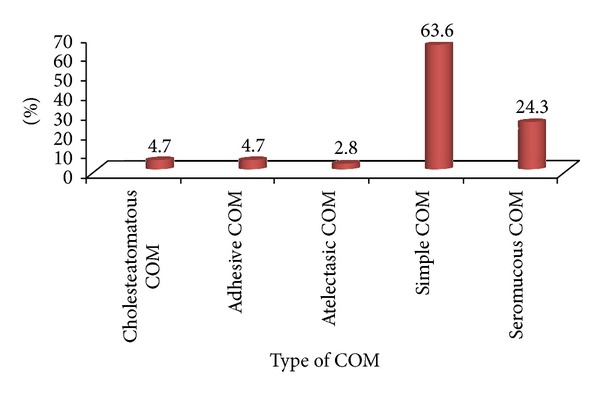
Distribution of cases by type of COM (*n* = 107).

The distribution of cases by type of COM was represented in Figure 3.

### 3.3. Paraclinical Data

Bacteriological examination was effective in 41 cases. Germs were found in 36 cases. There were 9 cases of *Staphylococcus aureus* (25.01%), 8 cases of *Pseudomonas aeruginosa* (22.20%), and 5 cases of *Proteus mirabilis* (13.90%). The rest of the germs (26.47%) were varied (*Streptococcus*, *Escherichia coli*, *Klebsiella,* and *Providencia*). No specific germ has been found. The sample was sterile in 5 cases.

 Antibiogram testing was performed in 31 cases. Among the antibiotics tested, ciprofloxacin and ceftriaxone were the most active, with respective rates in 26 cases or 83.90% and in 24 cases or 77.40% of cases.

The pure tone threshold audiometry was performed in all cases, but the types of hearing loss were identified in 76 cases (96.20%). Conductive hearing loss in 38 cases (50%) and mixed hearing loss in 32 (42.10%) were the most represented types in our series. This deafness was of varying intensity but dominated by the mild degree in 36 cases (47.37%).

### 3.4. Therapeutic and Scalable Data

Our patients received only medical treatment in 59 cases (74.68%): local antibiotherapy and anti-inflammatory treatment were systemically used, respectively, in 52 cases (88.13%) and 51 cases (86.44%). The treatment was medicosurgical in 20 cases (25.32%). It consisted mainly in a paracentesis (7 cases) in case of failure of medical treatment in the COM, a myringoplasty (6 cases), and a radical mastoidectomy in 4 cases of cholesteatoma.

The response to treatment was favorable with lesion stabilization in 63 cases (86.35%). Complications were observed in 7 cases (8.87%). They concerned mastoiditis in 3 cases (3.80%), peripheral facial paralysis in 3 cases (3.80%), and meningoencephalitis in 1 case (1.27%). Postotitis sequelae were dominated by deafness in 76 cases (96.20%) of variable intensity.

## 4. Discussion

In our experience, 79 patients with chronic otitis media were examined in 1 year, an amount of 0.96% of consultants. Abada et al. [3] in Morocco found 103 cases per year, Yeo et al. [4] (Seoul) 220 cases per year, and Osma et al. [5] (Turkey) 289 cases after 1 year of surveying.

Our rate seems low. Indeed, the actual frequency of this disease is difficult to specify in our conditions of exercise where health facilities, qualified staff, and technical facilities are limited [6]. Therefore, there is underreporting of cases in our context.

 The highest frequencies of COM cases were recorded during the period from March to June and during the month of December. This corresponds in Burkina Faso to the period of the dry, dusty Harmattan winds on the one hand and the cold on the other hand, that undermine the mucosa, favoring acute respiratory infections including acute otitis medium (AOM). It dominates the background with a rate of 73.41% of cases. It thus plays an important role in the genesis of COM [7, 8]. According to Triglia et al. [9] in France, the average length of the otitis episode in cold period is nearly three times longer than in warm weather. The seasonal influence is thus a significant factor in the genesis and maintenance of COM.

 The age group most represented in our study was that of 0–15 years with a frequency of 40.50%. These results are comparable to those of David and Peter [7] in the US, which recorded a rate of 39% for the age group from 0 to 15 years. We agree with these authors that at this age the immune immaturity of the child and the anatomical features of the ENT area predispose them to AOM and thus to COM. 

 The average delay for specialized consultation in our study was 6 months and 10 months for more than 25% of patients. This delay in specialist consultation could be partly accounted for by the lack of third-party payment (medical costs are entirely borne by the patient), and secondly, it is due to the importance of self-medication and the weight of traditional treatment in our context. To this could be added the low impact of the symptoms of this disease, which constitutes a trivializing factor in our regions.

Simple COM was the most represented in our series (71.02%) with a rather central perforation (61.73% of cases). These results are comparable to those of other authors such as Homøe et al. from Denmark [10] which noted a predominance of central perforation with a frequency of 86%. COM with effusion seems to be less frequent in our context (24.30% of cases). Nevertheless, many of this simple COM might be genuine COM with effusion seen at its superinfected and open stage.

Cholesteatoma COM accounted for only 4.70% of cases. These results tend to support a scarcity of cholesteatoma in our context. But this scarcity is relative for us since many cases certainly go unnoticed when you do not have a microscope for otology consultation. Moreover, in our developing countries, many patients, carriers of COM, do not consult ENT. And certain beliefs are still alive, “an ear which is flowing along hears very well.” Anyway, we must keep in mind the specific gravity of cholesteatoma COM [5, 11].

Among the contributing factors encountered, in-ear instillation of traditional products (46.09%) was the most represented followed by rhinitis (26.09% of cases).

 Rhinitis and nasopharyngitis, classic predisposing factors, are taken into account in the genesis of COM by their chronic impairment of tubal function [9]. However, in our context, we should first blame ear instillation of traditional products (46.09%) of badly known chemical nature. They will probably increase the risk of transition to chronicity and the development of extensive lesions. Unfortunately, the low purchasing power of patients, the influence of sociocultural habits (including pond baths), and disregard of the risks incurred induce this therapeutic use.

 The treatment of COM is medicosurgical [1, 6, 10, 12]. It not only helps to curb the infection process and the inflammatory process but also prevents otogenes complications. Topical antibiotics and anti-inflammatory are very important: 88.61% and 87.34%, respectively, of cases in our series. These results are consistent with the literature data [6]. Paracentesis and myringoplasty were the most practiced surgical interventions in our series followed by mastoidectomy. This surgical treatment concerned, respectively, four main directions: otitis media with effusion, simple open-spandrel COM, infectious complications, and cholesteatoma [7, 13]. The compulsory action in this last case remains a complete resection of cholesteatoma.

 The response to treatment is generally favorable, 86.35% of cases in our series. The early care and the quality of technical facilities are important assets for the prognosis [3, 6]. However, endotemporal complications are frequent in our series. The sequelae were primarily functional in our study, dominated by deafness with important socioprofessional consequences. The management of these sequelae is also a fundamental step in the management of COM.

## 5. Conclusion

Being a relatively common condition in our context, COM remains a public health problem that is often wrongly trivialized by people.

Inadequate technical platform creates diagnostic and therapeutic difficulties in developing countries like ours. We should undoubtedly focus on prevention through public awareness, an appropriate care of upper respiratory tract infections, and early treatment of AOM.

## Figures and Tables

**Table 1:** Distribution of patients according to ENT history.

Cases' history	Frequency	Percent
Acute otitis media	**58**	**73.42**
Otitis externa	9	11.4
Ear trauma	2	2.5
ENT tumors	2	2.5
ENT malformations	1	1.3
No ENT case history	**7**	8.9

Total	79	100

**Table 2:** Distribution of patients according to predisposing factors.

Predisposing factors	Frequency	Percent
Instillation of traditional products	**53**	**46.09**
Rhinitis	30	26.09
Pharyngitis	25	21.74
Sinusitis	2	1.74
Immunosuppression	2	1.74
Atopy	2	1.74
Diabetes	1	0.87

## References

[1] Baljosevic I, Djeric D, Milovanic J, Subarevic V (2008). Chronic suppurative inflammation of the middle ear in children. *Srpski Arhiv za Celokupno Lekarstvo*.

[2] Mostafa BE, El Fiky LM, El Sharnouby MM (2009). Complications of suppurative otitis media: still a problem in the 21st century. *ORL*.

[3] Abada RL, Mansouri I, Maamri M, Kadiri F (2009). Complications of chronic otitis media. *Annales d’Oto-Laryngologie et de Chirurgie Cervico-Faciale*.

[4] Yeo SG, Park DC, Hong SM, Cha CI, Kim MG (2007). Bacteriology of chronic suppurative otitis media—a multicenter study. *Acta Oto-Laryngologica*.

[5] Osma U, Cureoglu S, Hosoglu S (2000). The complications of chronic otitis media: report of 93 cases. *Journal of Laryngology and Otology*.

[6] World Health Organization (2004). Prevention of hearing impairment from chronic otitis media. *Report of a WHO/CIBA Foundation Workshop*.

[7] David P, Peter SR (2006). Middle ear chronic suppurative otitis medical treatment. *Otolaryngology*.

[8] Alho O-P, Oja H, Koivu M, Sorri M (1995). Chronic otitis media with effusion in infancy: how frequent is it? How does it develop?. *Archives of Otolaryngology*.

[9] Triglia JM, Roman S, Nicollas R Seromucous ear.

[10] Homøe P, Siim C, Bretlau P (2008). Outcome of mobile ear surgery for chronic otitis media in remote areas. *Otolaryngology*.

[11] Hua Z, Yan BH, Wai JT, Zhi KZ (2008). Clinical analysis of otogenic intracranial complications. *Annales d'Oto-Laryngologie et de Chirurgie Cervico-Faciale*.

[12] Diop EM, Diouf A, Ndiaye IC, Tending G, Tall A, Toure S Ear, nose and throat tropical diseases.

[13] Tabchi B, Rassi S, Elias R, Haddad A, Nehme P, El Rassi B (2000). Chronic suppurative otitis media. The experience of Hotel-Dieu Hospital. *Journal Medical Libanais*.

